# Porosity-Limited Transport during Two-Phase Surfactant/Polymer Floods in a Layered Sandstone

**DOI:** 10.1021/acs.energyfuels.4c04866

**Published:** 2025-01-23

**Authors:** Andrea Rovelli, Takeshi Kurotori, James Brodie, Bilal Rashid, Weparn J. Tay, Ronny Pini

**Affiliations:** † Department of Chemical Engineering, Imperial College London, South Kensington SW7 2AZ, U.K.; ‡ BP International Ltd, Chertsey Road, Sunbury-on-Thames TW16 7LN, U.K.

## Abstract

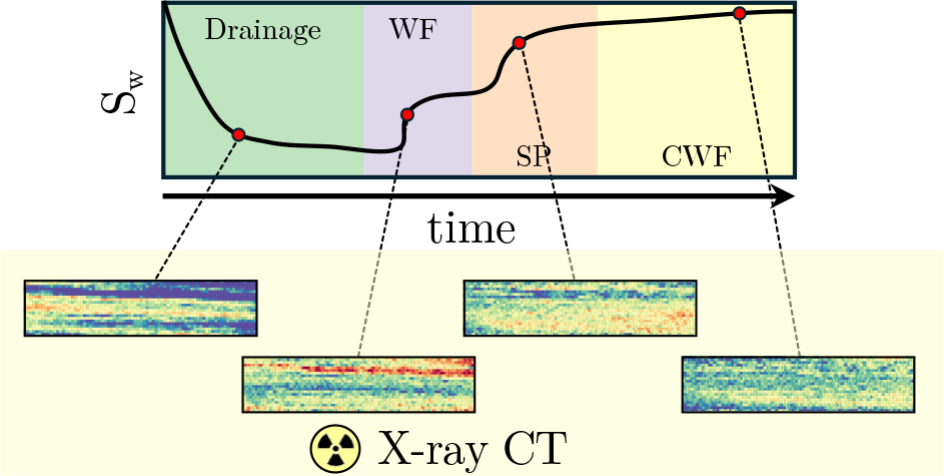

Surfactant/polymer flooding presents itself as an attractive technique for the full utilization of current reservoirs given its potential to yield high oil recoveries. Despite this appeal, discrepancies between laboratory and field results exist and limit their industrial implementation. Within the scale-up process, corefloods serve as a key tool for the evaluation of the recovery potential; however, due to complexities in the fluid system itself, these are commonly performed on homogeneous core samples. To further understand this, we conduct a surfactant/polymer flood as a tertiary recovery method within a Nugget sandstone core. A notable feature of the chosen core is its stratified nature, with layers of high and low porosity characterized via X-ray CT. Via the use of direct imaging, coupled with a step tracer test, preferential flow paths and slow-to-ingress regions of the core are identified, information that is then coupled with the surfactant/polymer flood results to better understand the mechanisms at play. To better understand the influence of the structured heterogeneity present within the core, the results are compared to an analogous experiment within a homogeneous sandstone core. We note the inability of an oil bank to form and the comparatively larger variability of the recoveries between different porosity layers within the core. Lastly, we highlight how, despite a high overall recovery of 80%, inefficiencies in the displacement process are still present and only observable due to the direct imaging methodology implemented, ultimately showcasing its value in this context.

## 1 Introduction

Surfactant/polymer flooding is a form of chemical enhanced oil recovery (cEOR) targeting improvements in both microscopic and macroscopic recovery factors.^1^ Despite the ongoing transition toward net zero,^2^ oil is still expected to play an important role in the energy system until late 2035.^3^ Following typical production from reservoirs, just 30% of the original oil in place is typically recovered.^4^ Enhanced oil recovery, and specifically cEOR, has thus always been of industrial interest^5^ given its potential to greatly increase the recovery from existing reservoirs, minimizing the need for additional exploration. Despite ongoing research, difficulties in translating lab-scale recovery factors to the field scale have been prevalent^6,7^ and, specifically for surfactant/polymer flooding, industrial application has thus been limited.^8,9^ Pivotal to any cEOR scale-up are laboratory corefloods. These experiments allow for both the evaluation of the oil recovery efficacy and the extraction of multiphase flow parameters used in reservoir models.^10^ Here, despite the prevalence of multiscale heterogeneity within reservoirs,^11,12^ studies have mainly focused on homogeneous media. This is a conscious trade-off to reduce the complexity of the system. There is already complexity in both the fluids used—such as microemulsions and chemical degradation^1^—and the coreflood interpretation itself^13,14^; the introduction of heterogeneity would further increase the complexity,^15,16^ ultimately resulting in a significant challenge for result interpretation.^17^

To expand the operating envelope of laboratory corefloods, direct imaging, and specifically medical X-ray CT, offers the ability to quantify local saturation development within multiphase experiments, allowing for a more comprehensive understanding.^18,19^ These include insights into the efficacy of the land trapping model in heterogeneous carbonate rocks^20^ and the role of microporosity in the capillary heterogeneity within rocks.^21^ Specifically, however, for surfactant/polymer flooding, we highlighted the numerous advantages of using X-ray CT to best interpret the corefloods in our previous work;^22^ however, like other studies on this subject,^23−25^ focus was on a homogeneous system. Within this work, we are thus primarily interested in investigating a structured heterogeneous system, specifically, a system comprising stratified layers. Due to their natural occurrence,^26,27^ studies within stratified systems are of importance; however, due to difficulties in sourcing or characterizing a naturally layered rock core, either sandpacks in series^28^ or artificial cores^29−31^ are often used as alternatives.

Inherently, their highly tunable nature allows for systematic investigations; particularly relevant is that of the impact of cross-flow on the oil recovery mechanism. Experiments omitting^32^ and including^33,34^ cross-flow are achievable and have ultimately pointed at an appreciable impact on the recovery mechanism and potential,^35^ observations further confirmed via modeling.^36,37^

Also key to most previous studies in stratified systems is the restricted viewpoint, given the lack of in situ imaging. As such, observations reported are “core averaged” and cannot investigate more detailed phenomena occurring within the porous media caused by the heterogeneity itself. Albeit limited, coreflood studies on stratified cores employing direct imaging exist and have proven extremely insightful, providing a compelling incentive for further research.^38−40^ Here, we extend the direct imaging methodology applied in our previous work on surfactant/polymer flooding^22^ to a system with a layered sandstone. In doing so, we are able to reconstruct the spatial variation of both porosity and saturation within a suitable error margin utilizing two-dimensional representations. To better understand the flow paths within the core, we conduct a step tracer test and identify the presence of slow-to-access, low-porosity, regions. The surfactant/polymer flood is then performed as a tertiary recovery method and, by analyzing the spatial variation of the saturation, we highlight key in situ behaviors induced by the structured heterogeneity of the system.

## 2 Materials and Methods

### 2.1 Materials

For the OP, a model oil represented by decane (≥95%, Sigma-Aldrich, CAS: 30570) was used. For the AP, the brine salinity was altered using sodium chloride (≥99.5%, Sigma-Aldrich, CAS: 31434-M) and set to 3.5 wt % NaCl so as to match the optimal salinity for the surfactant/polymer solution. The surfactant/polymer was a 1 wt % L-145–10s 90 (90% active, Sasol) and 1500 ppm of HPAM (FP3530s, SNF Floerger) formulation. Again, the salinity was 3.5 wt % NaCl as it was found to be the salinity at which the interfacial tension was minimized.^22^ The viscosities are 1, 0.85, and 25 mPa s for the brine, oil, and surfactant–polymer solutions, respectively. The tracer used in the experiment was 3.8 wt % NaI in water, selected to both approximately match the density of the brine and thus minimize any buoyancy effects and provide a strong X-ray CT contrast due to the dopant nature of NaI. Lastly, to inject the surfactant/polymer solution into the core, light mineral oil (Sigma-Aldrich, CAS: 330779) was used as the displacing fluid. This combination of fluids was found to both yield excellent recoveries and form an oil bank when used on Bentheimer sandstone in our previous work.^22^

**Figure 1:**
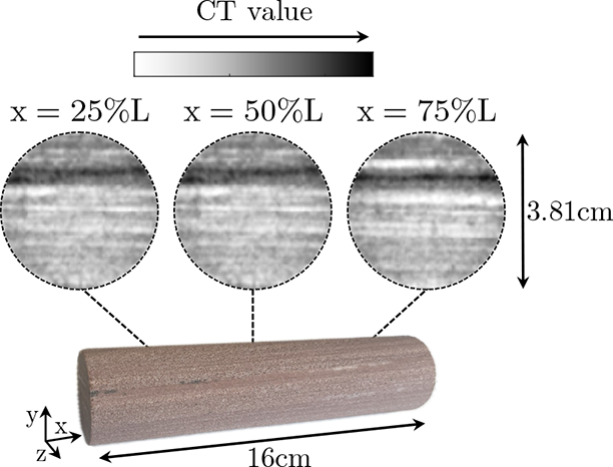
Showcase of the Nugget sandstone core used within the experiment. Shown are both a photo of the core itself and images of three cross sections of the core acquired utilizing medical X-ray CT. Within the scans, the darker colors refer to regions with stronger X-ray attenuation.

For the rock, a Nugget sandstone of 16 cm length and 1.5 in. diameter (Kocurek Industries Inc.) was used. Nugget is an eolian sandstone characterized by stratas.^41^ The stratas themselves result from both variations in grain sizes—typically fine—and variations in grain sorting, as a consequence of the depositional origin.^42^ The core used here is stratified in nature with altering layers, in the longitudinal-axial plane, of different porosities, shown in Figure 1. The average porosity of the sample was found to be 11.05%, yielding a pore volume (PV) of 20.16 mL. The permeability was estimated as 36.51 (±0.82) mD from a multirate steady-state test with brine and analyzed using Darcy’s law; full details are given in the SI.

### 2.2 Experimental Setup

**Figure 2:**
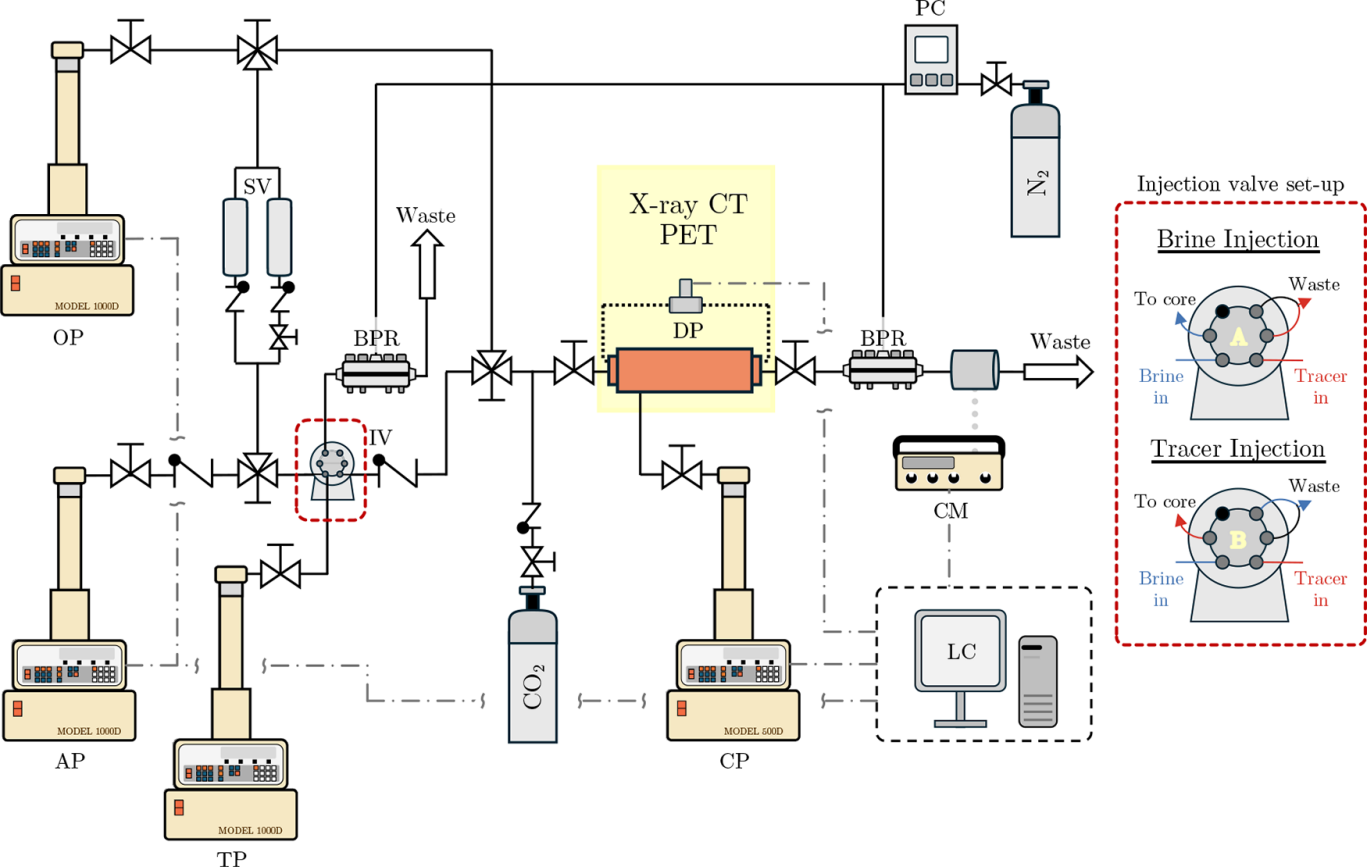
Diagram of the coreflooding experimental setup used. Also shown is the approach used with the six-port inlet valve to allow for the alternation between injection of brine and tracer without interruption of flow. See the manuscript text for a description of the various components.

The experimental system used in this work is a modification of that from Rovelli et al.^22^ and is shown in Figure 2.

The rock core is housed in an aluminum coreholder and placed within the medical X-ray CT scanner (Toshiba Aquilion 64 TSX-101A). To inject fluids, three piston syringe pumps (1000D, Teledyne ISCO) are used: one for the injection of the aqueous phase (AP), one for the oleic phase (OP), and one for the tracer solution. An additional piston syringe pump (CP, 500D, Teledyne ISCO) is used to impose a confining pressure on the rock to avoid any fluid bypass. The pore pressure within the core and the bypass waste line are held using two back-pressure regulators (ZF series, Equilibar) connected to a pressure controller (EPR 3000, Equilibar) and a nitrogen cylinder. Also, upstream of the coreholder is a dual-position six-port valve (IV, Cheminert 6 port injection valve, Vici) that allows for the rapid switching between the AP (brine) and the tracer. Through this approach, both the brine and tracer can be continuously flowed at the set pore pressure, with one going to a bypass waste, and the injecting fluid can be quickly switched with minimal disturbance to the system. Also, upstream of the coreholder are two sample vessels (304L SS Sample Cylinder, Swagelok) housing the surfactant/polymer solution, which is then injected via displacement using mineral oil. Downstream of the coreholder are a conductivity cell and a meter (CM, Model 8032, Amber Science) for conductivity measurements. In addition to all of the pump flow rates, volumes, and pressures, the differential pressure over the core is measured (DP, 3 bar PRD-33X, Keller UK) and logged utilizing a logging computer.

### 2.3 Experimental Methodology

#### 2.3.1 Solution Preparation

The brine, tracer, and surfactant/polymer solutions were made utilizing 18 MΩ deionized water and the addition of the associated components/salts, mixed until full dissolution was achieved. Prior to injection into the core, the solutions were filtered using 0.45 and 1.20 μm pore size filters (MF-Millipore membrane filters, Sigma-Aldrich, CAS: HAWP04700 and RAWP04700) for the brine/tracer and surfactant/polymer solutions, respectively. All other fluids were used directly as purchased.

#### 2.3.2 Coreflooding

The coreflood was performed at room temperature, 21.3 (±1.1) °C, and throughout the experiment, a confining pressure of 30 bar was set. Prior to mounting within the coreholder, the rock was dried at 65 °C for 72 h in an oven. Once mounted, the core was flushed with CO_2_ (≥99%, BOC) to displace any air and allow for complete water saturation of the rock. Brine was then injected, and the pore pressure was set to 8 bar via the back-pressure regulator. Permeability was measured, and a tracer test at 2 mL/min was conducted where the tracer, NaI, was continuously injected for approximately 10 PV, following which brine was injected in an identical fashion for approximately 10 PV, with scans acquired during both the tracer and brine injections. Drainage was then performed via the injection of decane at 1.5 mL min^–1^ for at least 20 PV, and a waterflood was then performed. The waterflood was run overnight to completion, determined via monitoring of the pressure drop along the core, and was subsequently followed by the surfactant/polymer flood, where the surfactant/polymer formulation was injected continuously for approximately 1.5 PV. Lastly, a final waterflood, chase waterflood, was started and run until termination of the experiment. For all injection steps following the drainage, the chosen flow rate was 0.2 mL/min, which corresponds to a frontal advance rate of 7 ft/day^–1^. This was selected to lie within representative field scale values^43^ while also allowing for a practical, time-wise, experiment.

### 2.4 Image Processing and Analysis

The primary data acquisition method used within this experiment was via medical X-ray CT scanning. As such, scans were obtained throughout the aforementioned experimental steps. In general, at the start of new injection steps, where the system is most dynamic and the rate of change is greatest, scans were obtained with the highest frequency, typically every 0.05 PV. The scan time was 10 s, corresponding to 0.017 PV and 0.002 PV for the fastest and slowest flow rates, respectively. Scans were then obtained sparsely throughout the continuation of the injection steps to monitor the progression of the experiment.

Following acquisition, raw scans can be combined to calculate porosity,^44^ ϕ, and oil saturation,^45^*S*_o_, from the following expressions:


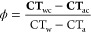
1


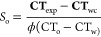
2

Here, **CT**_exp_ is the CT number of the experimental scan of interest, **CT**_wc_ is the CT number of the water-saturated scan of the core, and **CT**_ac_ is the CT number of the air-saturated scan of the core. The voxel-, slice-, or core-averaged porosity and saturation values are then obtained using the voxel-, slice-, or core-averaged CT numbers. CT_w_, CT_o_, and CT_a_ are the constant CT numbers of pure water, oil, and air within the coreholder, respectively. These were determined by taking scans of the fluids within a test tube placed into the coreholder and were calculated as 144.71, −142.49, and −833.86 HU, respectively.

Importantly, eq 2 can also be applied to calculate the concentration of the tracer, *c*_NaI_, by substituting CT_o_ with CT_NaI_, which is calculated as 653.56 HU.

Error quantification can be performed via propagation of variances,^46,47^ and it can be shown that the absolute error in the oil saturation, eq 2, can be given as
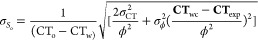
3where σ_ϕ_, the error in porosity, is calculated from
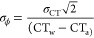
4

and σ_CT_ is obtained via the acquisition of repeated scans, the calculation of their differences, and the subsequent quantification of the random error associated with the CT scanning. The raw scans acquired have voxel sizes of (0.122 × 0.122 × 1) mm^3^; however, it was found that, due to the low porosity and undoped brine/oil combination, in order to obtain reasonable error values, resampling of the images was necessary. Due to the stratified nature of the rock investigated, it was decided to resample the images to (1.95 × 1.95 × 1) mm^3^ and then average the core in the longitudinal direction, yielding a two-dimensional image. This minimizes the absolute error—σ_*S*_0__ = 8.7 × 10^–2^, σ_NaI_ = 4.8 × 10^–2^, and σ_ϕ_ = 2.8 × 10^–3^—while preserving the most valuable information by avoiding coarsening in the axial direction. Where possible, to further reduce the error, repeated scans were taken, shown to reduce the error by , where *n* is the number of repeated scans.^48^ Calculations of errors associated with different coarsening schemes are given in the SI.

Lastly, all of the two-dimensional visualizations presented within this work were generated using a framework built upon the MATLAB Reservoir Simulation Toolbox.^49^

## 3 Results

### 3.1 Core Porosity

**Figure 3:**
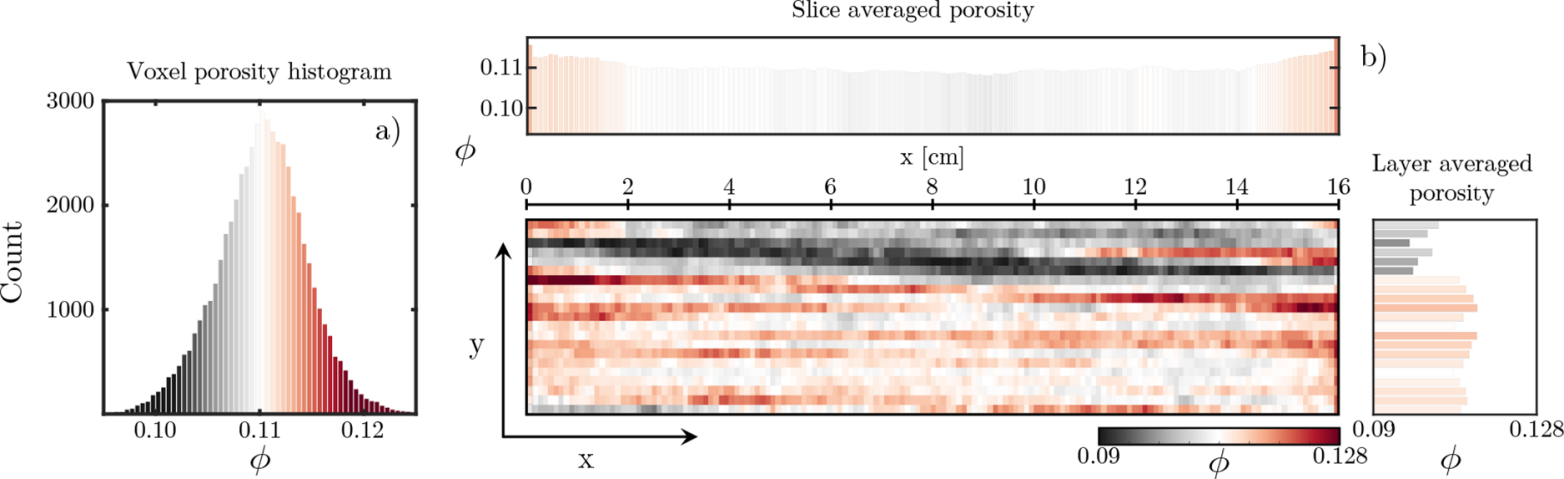
Porosity characterization of the Nugget sandstone core used within the experiment. (a) Histogram of voxel porosities extracted from three-dimensional reconstruction of scans. (b) Spatial variation of porosity within the core. Shown are the two-dimensional representation, the slice-averaged porosity, and the layer-averaged porosity.

A key feature of the Nugget sandstone core used in this experiment is its stratified nature. This characteristic is a result of layers of differing porosities and, to further investigate this, Figure 3 presents an overview of the porosity characteristics of the core.

Shown in Figure 3a is the histogram of the voxel porosity within the core extracted from a three-dimensional representation with voxels of size (1.95 × 1.95 × 1) mm^3^. Note, given the use of repeated scans, the absolute error for the porosity is low even in the three-dimensional representation, calculated as σ_ϕ_ = 4.5 × 10^–3^. The distribution itself appears normal and centered around the mean value of 11.08%. The range of porosities displayed is also narrow, and most voxels lie between 10 and 12% porosity. Figure 3b, on the other hand, shows the spatial variation of the porosity itself. The two-dimensional map illustrates the clear existence of layers of differing porosity values. Notably, at the top of the core, a large low porosity layer exists for the full extent of the core. Directly below this, regions of higher porosity are also seen spanning sections of the core. Another lower porosity layer appears to exist toward the outlet three-fourths down the core. One can better see this variation in porosity between layers by examining the layer-averaged porosity bar graph, emphasizing both the low porosity regions at the top of the core and the relatively higher porosity layers toward the core center. Because of the known proportionality between porosity and permeability in sandstones,^50^ we expect these layers will also transport fluids at different velocities. Lastly, from the slice-averaged porosity bar graph, we note a deceptively homogeneous profile, once again centered around the mean of 11.08%.

### 3.2 Tracer Test

**Figure 4:**
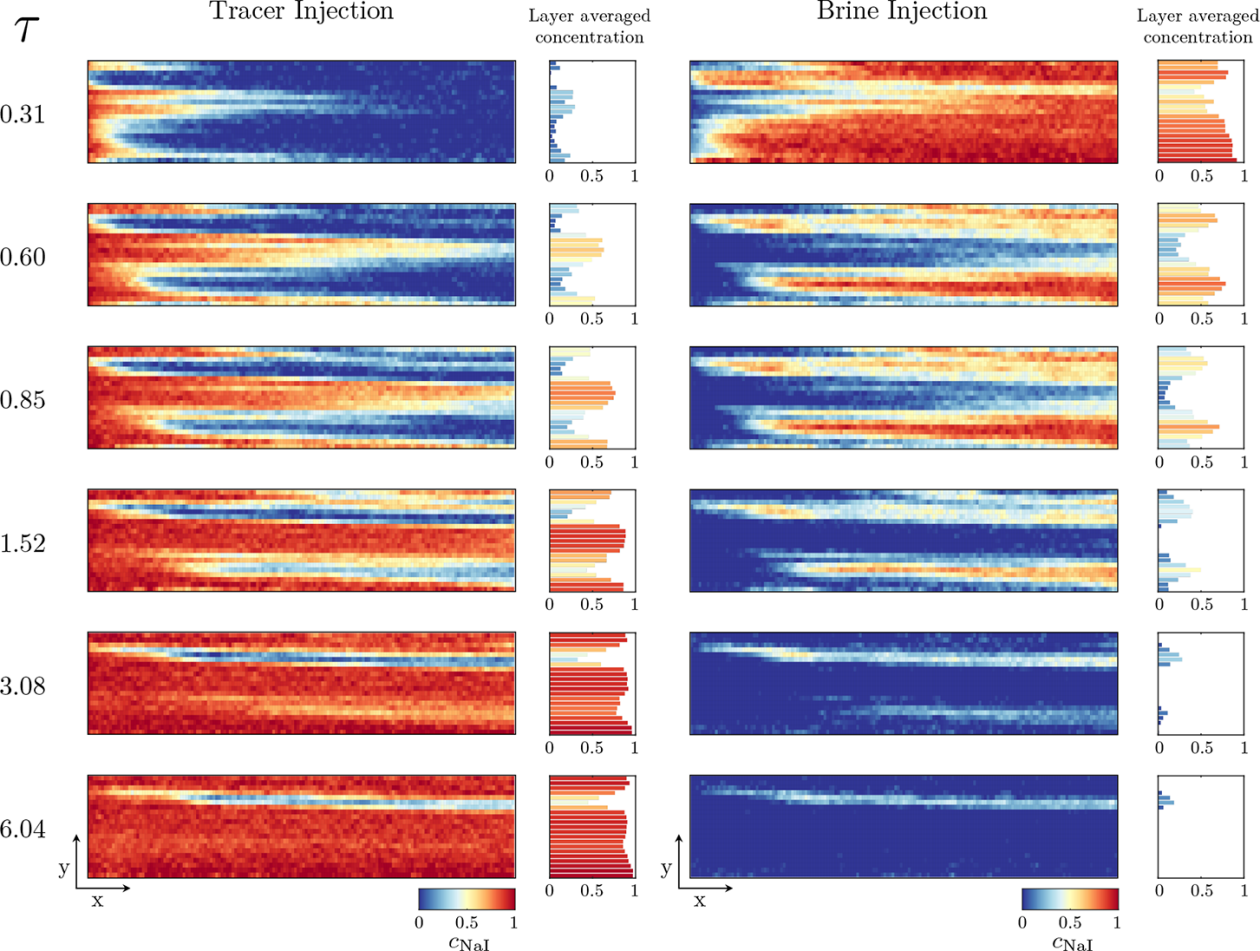
Two-dimensional representations of the tracer tests conducted on the Nugget sandstone. Shown are both the results associated with the injection of the tracer, NaI (left), and the brine (right). Time or pore-volume injected, τ, increases downward, and the concentration of tracer is given by the colormap, with red indicating a high NaI concentration. For each two-dimensional representation, the averaged concentration of tracer within each layer is also shown.

Although all steps of the injection stages were imaged, as the focus of this work is the efficacy of the surfactant/polymer flood applied to a stratified rock system, the results focus on the tracer test conducted, to yield insights into the preferential flow paths, and the surfactant/polymer flood itself. For the tracer test, Figure 4 provides a visual representation of both the tracer injection and the subsequent, analogous, brine injection.

Within Figure 4, both the two-dimensional representations of the evolution of the tracer concentration and the layer-averaged tracer concentrations are shown for increasing time steps. Time is reported as PVs injected, τ, and calculated as τ = *Q*/PV_core_, where *Q* is the volumetric injection flow rate and PV_core_ is the PV of the core. The imaging results allow for a number of important observations regarding the preferential flow paths present within the rock. First, the rapid invasion, and breakthrough, of the tracer within the middle layers is evident. The bottom and top of the core are the next to be invaded, with the top layers appearing more hindered toward the outlet side of the core. Two comparatively less permeable layers are also evident: one at the bottom, toward the outlet, of the core and one spanning a large portion of the top of the core. In both regions, the tracer is slow to invade, with both layers seeing minimal tracer ingress by 1.5 PV, and, specifically for the top region, low ingress even after 6 PV. Importantly, these observations are also evident in the subsequent brine injection, with profiles that closely resemble those from the tracer injection, yielding confidence in the observations being indicative of the true behavior of the system.

From the behavior observed and the results relating to the porosity of the core (Section 3.1), the comparatively less permeable regions can be mapped to the low porosity regions present within the core. This is an important observation because, as aforementioned, the distribution of porosity within the core is narrow, as seen in the histogram in Figure 3. Hence, the variations of the porosity between the layers are also minor; however, as can be seen from the results in Figure 4, the apparent impact on the preferential flow paths is major and cannot be neglected.

**Figure 5:**
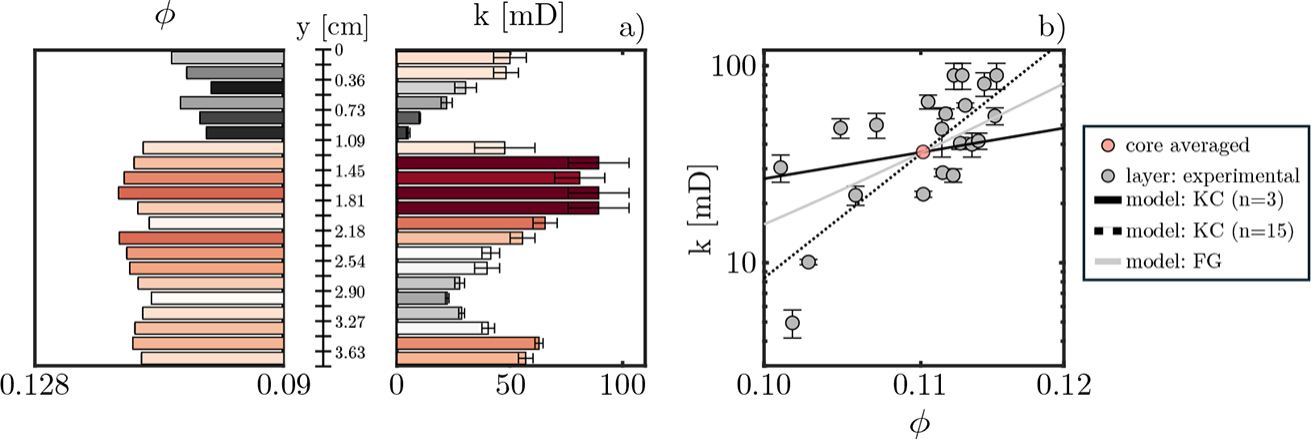
Porosity–permeability relationship for each layer within the Nugget core. Permeabilities are estimated from both step-up and step-down experiments (bounds in error bars); bars and markers show the mean. (a) Spatial comparison of the relationship. (b) Numerical comparison of the relationship. Shown here are *k*(ϕ) models (lines): Kozeny–Carman (KC) and fractal geometry (FG).

To further investigate the porosity–permeability relationship, an estimate for the layer permeabilities can be made by considering the arrival time,^51^ and thus effective velocity, of the tracer within each layer^52^; full details have been outlined in the SI. Through this, the porosity–permeability relationship of each layer can be visualized as is done in Figure 5.

In Figure 5, the permeabilities for each layer are estimated from both the step-up and step-down tracer tests, and their resulting average is shown. Examining the spatial distribution of the permeability in subfigure (a), one can better note the more pronounced variations on a layer–layer basis compared to the porosity. Specifically, despite minor variations in porosity in the lower portion of the core (*y* ∈ [2.18, 3.63] cm), their impact on the apparent permeability of the corresponding layer is comparatively pronounced. To further investigate the extent of this relationship, subfigure (b) presents ϕ-*k* for each layer. Here, once more, one can note that, despite the relatively small variations in the porosity, the estimated layer permeabilities are significantly different—note the logarithmic *y*-scale—implying a high-order porosity–permeability relationship. Such a relationship can be investigated via porosity–permeability models. Used here are the KC model,^50^ with two different porosity exponents, *n*, and an FG^53^ model—both models outlined in the SI. These models are applied to predict the permeability from the experimental porosity data, constrained in mean permeability by that calculated for the core, the results of which are also shown in Figure 5. From the model predictions, the key observations are the poor scaling observed from the traditional, *n* = 3, KC model and the improved accuracy from both the higher order KC model (*n* = 15) and the FG model, a model with a naturally higher order porosity–permeability relationship. Overall, the results from both Figures 5 and 4 highlight how, despite the comparatively minor variations in layer porosity, the underlying relationship to the permeability and, as such, the preferential flow paths is of a high order and can be expected to contribute measurably to any in situ behavior.

### 3.3 Surfactant/Polymer Flood: X-ray CT Imaging

**Figure 6:**
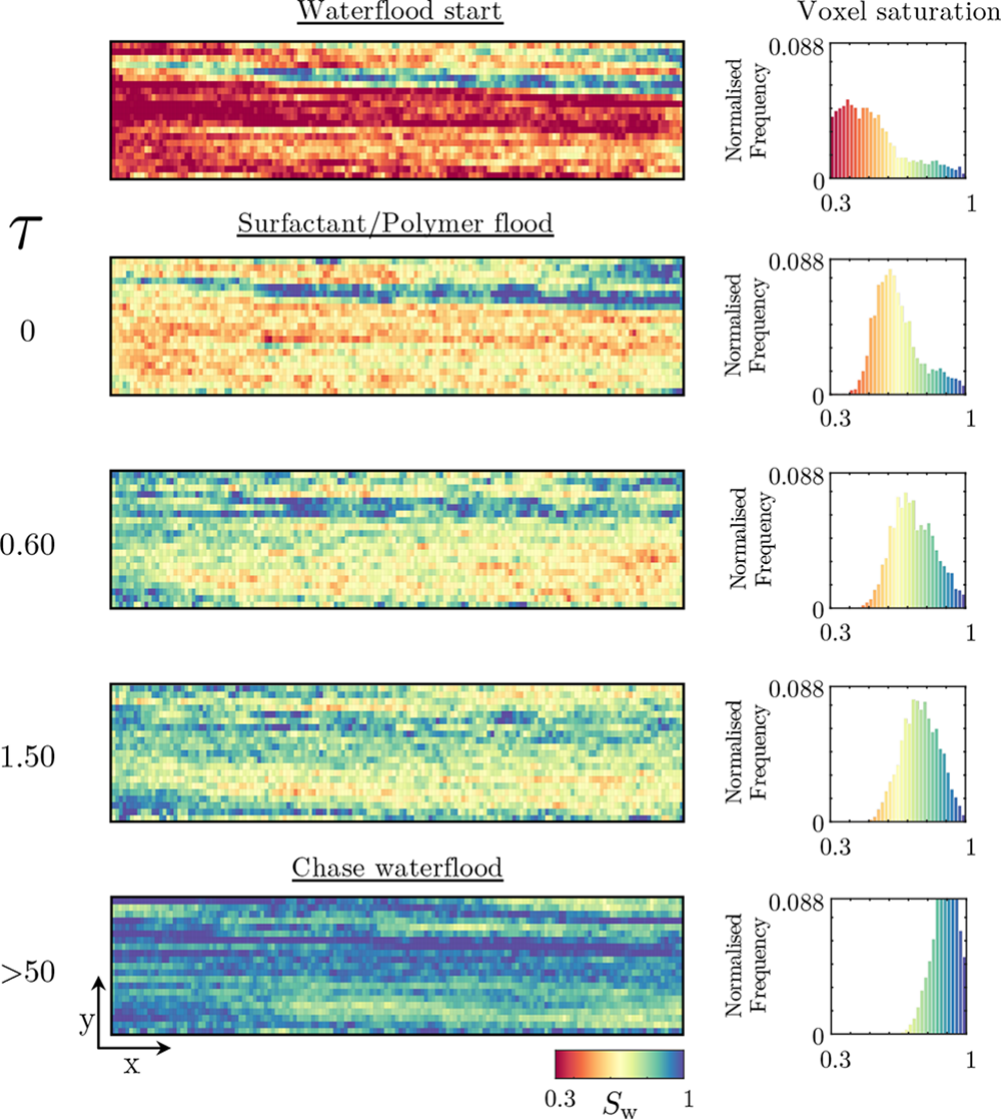
Two-dimensional representations of the surfactant/polymer flood conducted on Nugget sandstone; the flooding direction is from the left to the right. Time or pore-volume injected, τ, increases downward, and the saturation of water, *S*_w_, is given by the colormap, with blue indicating high water saturation. For each two-dimensional representation, the histogram for voxel saturations is also given.

Applying an identical imaging approach to the surfactant/polymer flood, Figure 6 presents the evolution of the water saturation within the core in both a 2D map and a histogram of the water saturation within the system.

Prior to examining the surfactant/polymer flood itself, via the two-dimensional representations of the water saturation, one can first examine both the conditions following the drainage of oil (injection of oil within the water-saturated core) and the conditions following the completion of the subsequent waterflood. The conditions at the end of drainage, top profile in Figure 6, showcase how, despite the injection of ≥20 PV of oil, the tight layer at the top of the core remains, relatively, water-saturated, additionally seen from the associated histogram where a number of voxels remain at high water saturations. This could be considered an expected result as, in addition to having a lower permeability, it is highly likely that the capillary entry pressure of the tighter regions is higher. Furthermore, when performing the drainage, as the oil saturation within the high porosity regions increases, so does the oil relative permeability, leading to these layers increasingly dominating the oil flow path. The profile following the waterflood, the initial state of the surfactant/polymer flood, shows dependent results. The center layers, more permeable regions, contain most of the trapped residual oil with the low porosity regions containing a disproportionately high water content due to both the aforementioned initial state of the waterflood and the favorable recovery in these regions given the core’s water-wet nature. Once the surfactant/polymer flood commences, the water saturation within the core appears to systematically increase in a fairly uniform fashion; in fact, as will be discussed in a later section (Section 4), a notable feature not present within this surfactant/polymer flood is the formation of an oil bank—this is despite our previous work demonstrating this formulation’s capability in doing so.^22^ Instead, by examining the voxel saturation histograms, we note a slow transition of the distribution to a bell shape, followed by an increase in the mean water saturation as the experiment progresses. Overall, both from the two-dimensional maps and from the histograms, the surfactant/polymer flood clearly achieves an additional recovery; however, it appears to do so by first homogenizing the saturation within the core and then gradually increasing its value, undesirable from an efficiency point of view.

A final key aspect evident from the imaging results is the final state of the spatial distributions of the voxel saturations. Unlike the initial state, where the comparatively less permeable layers contained a disproportionately high water content, the opposite now appears true. Following the completion of the surfactant/polymer flood, and associated chase waterflood, most of the final residual oil appears trapped within the low porosity layers—an important observation to be discussed further in Section 4.

### 3.4 Analysis of the Surfactant/Polymer Coreflood

**Figure 7:**
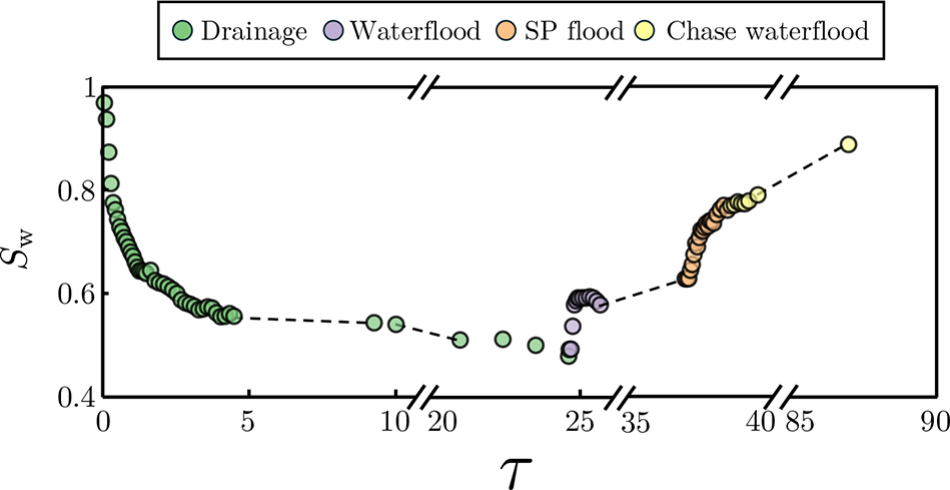
Evolution of overall water saturation, *S*_w_, throughout the whole coreflooding experiment. This is given as a function of the PV injected, τ, and can be split between the injection steps: drainage, waterflood, surfactant/polymer (SP) flood, and the chase waterflood.

To complete the overview of the generated data set, Figure 7 presents the evolution of the overall water saturation within the core for the whole duration of the experiment starting from the drainage.

The overall saturation presented here is calculated via the acquired scans and the application of eq 2. In terms of experimental overview, the drainage initially sharply decreases the water saturation and then slowly plateaus following 5 PV injected, ending at an average water saturation of 48.3%. The following waterflood raised the overall water saturation to 65.6%, yielding a recovery of 33.6%. The surfactant/polymer flood further increased the saturation to 77.0% over the 1.5 PV injected, corresponding to an incremental recovery of 33.2%. Lastly, the chase waterflood, run to completion, yielded a final water saturation of 88.9%, an incremental recovery of 51.7% from the end of the surfactant/polymer flood. This final incremental recovery is especially high and, as will be discussed in the next section, can most likely be attributed to the layered nature of the core and the associated preferential flow paths.

## 4 Discussion

To best understand the results obtained, it is useful to compare them to a more idealized system. For this, we can utilize results from an analogous experiment conducted on a Bentheimer sandstone core of 15 cm length and 1.5” diameter from Rovelli et al.^22^ The fluids and injection strategy employed were identical; however, due to the differing mean porosity, 23.7% in the Bentheimer core, the flow rates for the injection steps differ between the rocks to maintain an identical frontal advance rate.

**Figure 8:**
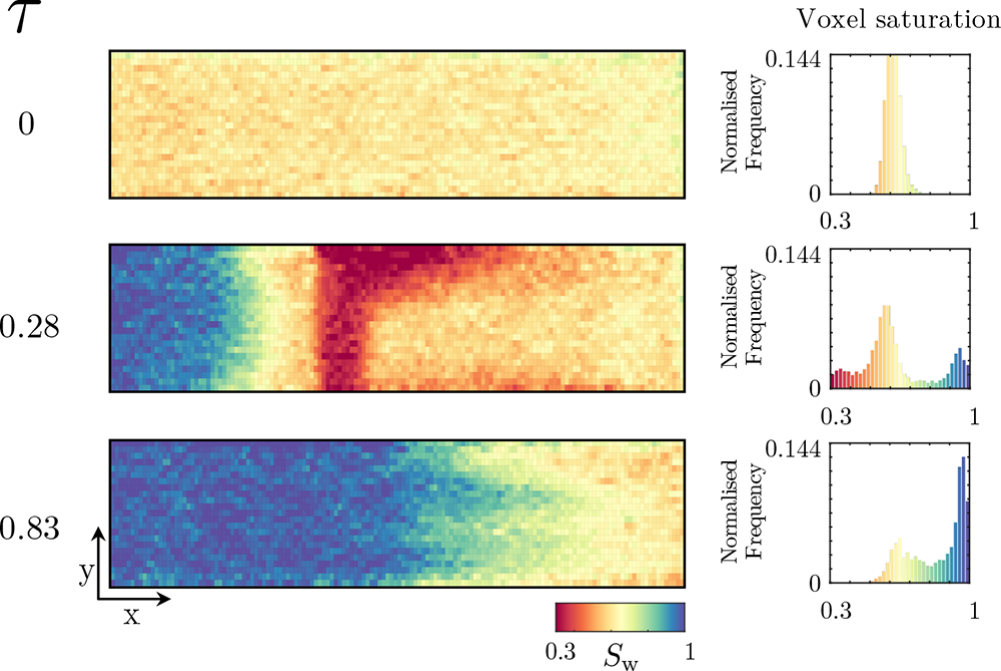
Two-dimensional representations of the surfactant/polymer flood conducted on Bentheimer sandstone; data from Rovelli et al.^22^ Time or pore-volume injected, τ, increases downward, and the saturation of water, *S*_w_, is given by the colormap, with blue indicating high water saturation. For each two-dimensional representation, the histogram for voxel saturations is also given.

Both Bentheimer and Nugget are sandstones with similar compositions, with a quartz content of over 90%^54,55^; as such, differences introduced by any rock/fluid interactions are minimized. The key difference between the two experiments is thus the existence of structured heterogeneity within the Nugget sandstone core. Aforementioned in Section 3.3 is the lack of an oil bank formed within the surfactant/polymer flood; see Figure 6. To exemplify this observation, Figure 8 presents an identical representation of the analogous surfactant/polymer flood on Bentheimer.

**Figure 9:**
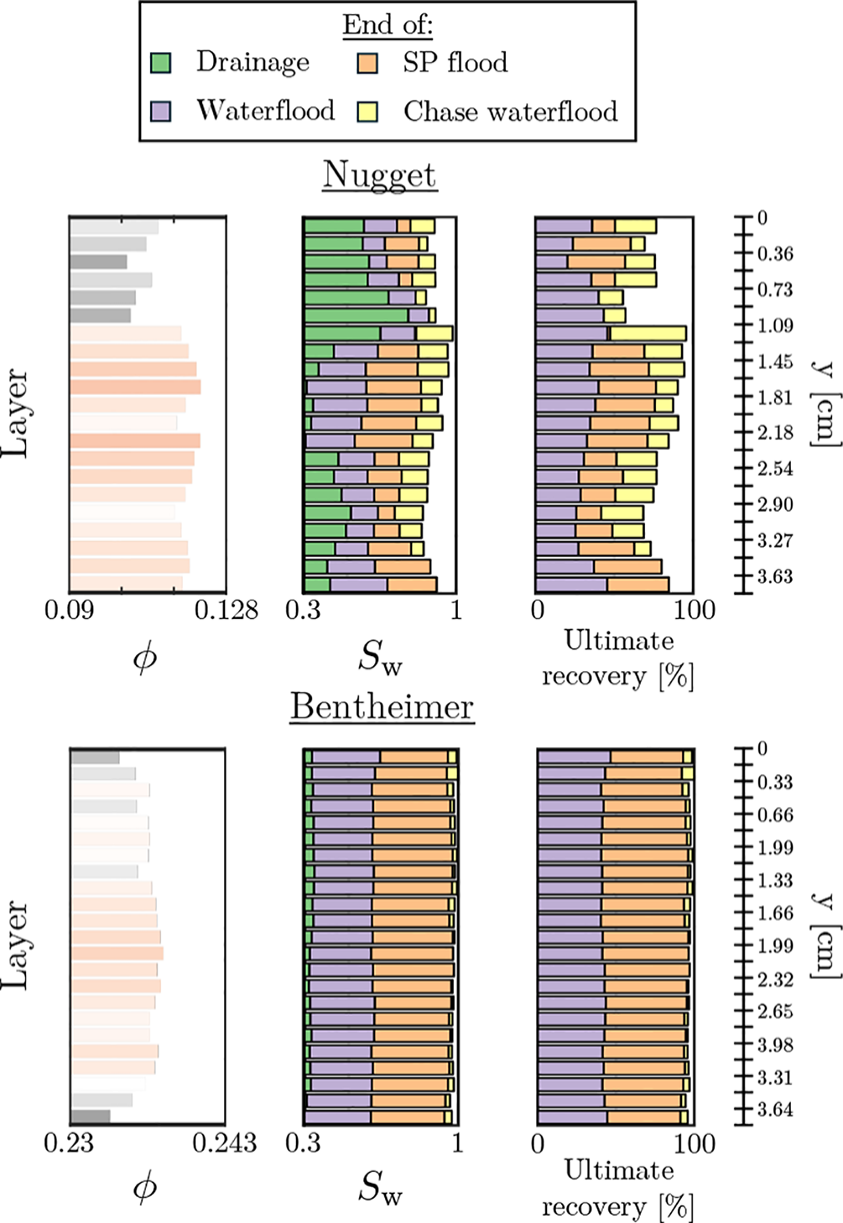
Comparison of identical experiments conducted on both Nugget sandstone (presented here) and Bentheimer sandstone (data from Rovelli et al.^22^). The figure depicts a layer-averaged interpretation, where the porosities of the rock, saturations during the experimental progression, and oil recoveries are shown.

Contrasting the surfactant/polymer flood in the Nugget core, Figure 8 shows the clear formation of an oil bank in the Bentheimer experiment. This is evident in both the two-dimensional representations, with a region of high oil saturation moving through the core, and the voxel saturation histograms, with a trimodal distribution. Specifically, the trimodal distribution contains three peaks: one for the oil bank—low water saturation, one for the region swept following the oil bank—high water saturation, and one for the initial water saturation—intermediate saturation. As the oil bank progresses through the core, the transition between the low and high water saturations is rapid and indicative of an efficient recovery from the surfactant/polymer flood on a voxel-by-voxel basis. This is in clear contrast to the slow transition in mean water saturations seen in Figure 6 for the Nugget core. Given the identical fluids and injection strategy, the inhibiting nature toward the formation of an oil bank must lie in the layered nature of the Nugget core. To best investigate this, Figure 9 presents a layer-based viewpoint of the comparison between the two rock types.

**Figure 10:**
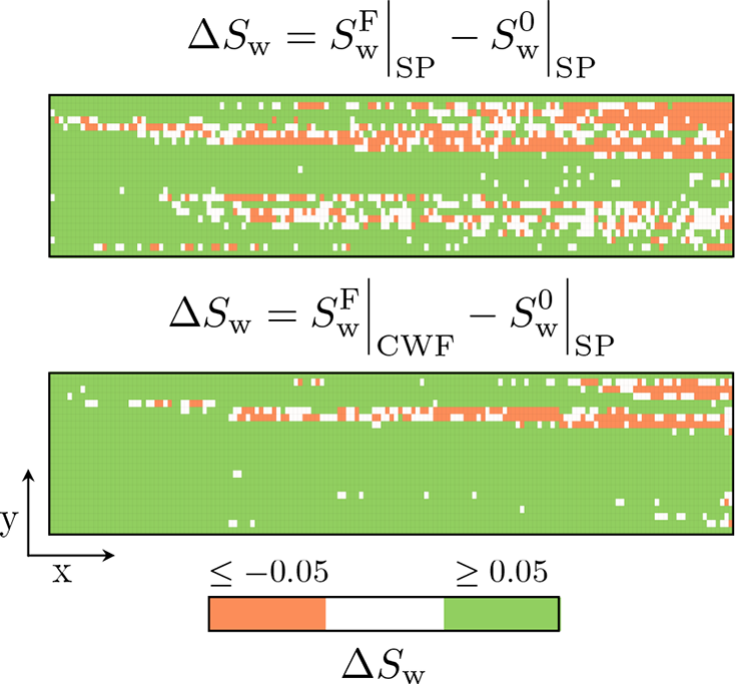
Two-dimensional representation of the change in saturation from the start of the surfactant/polymer flood (SP) to the end of the surfactant/polymer flood (top) and chase waterflood (CWF, bottom). Highlighted are the regions with a noticeable increase in the water saturation (green) and regions with a noticeable decrease in the water saturation (orange).

Figure 9 presents the layer-averaged porosities, the layer-averaged water saturations at the end of the key injection steps, and the layer-averaged recoveries from the injection steps; this is done for the experiments on both the Nugget and Bentheimer cores. Evident from the first observation is the homogeneity in the Bentheimer results. Following all injection steps, the water saturation between layers is invariant; accordingly, the recoveries from the injection steps are also invariant between layers. This is in stark contrast with the results for the Nugget core, where large differences exist on a layer-by-layer basis. Specifically, key differences exist between the low porosity layers at the top of the core and the central, comparatively high porosity, layers. For the high porosity layers, the behavior is similar to that of Bentheimer, with fairly homogeneous results displayed in the central layers. More notable, on the other hand, are the results for the low porosity layers. From Figure 3, and the preferential flow paths of Figure 4, two primary regions of low porosity were identified: a larger layer at the top of the core and a region toward the bottom, outlet side, of the core. Examining the recovery at the end of each injection step within Figure 9 for these layers, we note the following. For the low porosity region at the top of the core (*y* ∈ [0.73, 1.09] cm), the surfactant/polymer flood appears to have no impact on the layer-averaged saturations compared to both waterfloods. For the region at the bottom of the core (*y* ∈ [2.90, 3.27] cm), however, a measurable recovery from the surfactant/polymer flood is seen, and a larger recovery from the chase waterflood is also observed. To best investigate the behavior observed, Figure 10 presents a binarized spatial variation of the absolute change in saturation between the start of the surfactant/polymer flood and both the end of the surfactant/polymer flood (top panel) and the end of the chase waterflood (bottom panel).

Of primary interest within the two-dimensional representations are the regions where the surfactant/polymer flood had no impact (white areas) and those where the surfactant/polymer flood had a negative impact (orange areas) with an increase in oil saturation of ≥0.05. The decision for these thresholds is based on propagating the error in the saturations considering repeated scans to ensure an accurate representation of the system (see the Supporting Information section “Error Propagation and Correlation”). From Figure 10, it is evident that the locations in which an increase in oil saturation occurred are the low porosity areas aforementioned. Notably, the observed local saturation changes within these layers contrast with the absence of changes when layer-averaged saturations are considered (refer to Figure 9). The latter are in fact the result of the specific local saturation distribution (i.e., as much oil has been produced from these layers as it has been introduced due to, e.g., cross-flow). However, it is also clear that the two low porosity regions operate on different time scales in terms of recovery given the difference in the spatial map following the chase waterflood.

Comparing the state at the end of the chase waterflood and that at the end of the surfactant/polymer flood, the low porosity region at the bottom of the core sees an increase in the water saturation as it transitions from an area with no impact to an area with a positive impact compared to the surfactant/polymer flood start. This is in contrast to the low porosity region at the top of the core, which remains an area with a negative impact. Two events can thus be considered of interest: one is the cause of the negative impact and the other is the difference in recovery between the low porosity regions.

For the first consideration, due to the complexities of the phenomena present within surfactant/polymer flooding, attributing a certain cause is difficult; however, a possible interpretation is the combination of two contributing factors. The first is the surfactant’s ability to significantly reduce the interfacial tension. This decrease in interfacial tension has been shown to correlate with an increase in relative permeability at a fixed water saturation—and a loss of curvature in the relative permeability curves themselves^56^—which, in combination with the decrease in capillary entry pressure due to the reduction in interfacial tension, would ultimately aid in the displacement of the decane within the tighter regions of the core. The second is the large increase in the pressure drop during the surfactant/polymer flood due to the injection of a viscous fluid, with a peak pressure drop of about 5 times that of the waterflood; the profiles are shown in the SI. Combined, the capillary/viscous force balance was shifted, as captured by the capillary number,^57^*N*_c_ = *K*Δ*P*/σ, and led to the ingress of oil within the tighter regions of the core.

**Figure 11:**
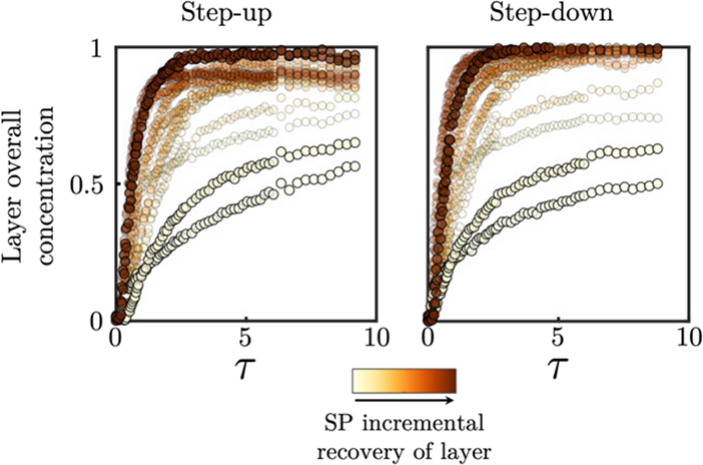
Temporal evolution, as a function of PV injected (τ), of the overall concentration of tracer (or brine) within the individual layers during the tracer (left panel, “step-up”) and brine (right panel, “step-down”) injection, visually represented in Figure 4. The individual curves for each layer are mapped to the incremental recovery from the surfactant/polymer (SP) flood through the colormap (data extracted from Figure 9). This is further emphasized by reducing transparency on all curves but the two curves with both the highest and lowest SP incremental recovery. For clarity of presentation, the recovery values are relative and scaled.

The second consideration regarded the difference in recovery from the two low porosity regions at the top and bottom of the core during the chase waterflood. This could ultimately be attributed to the short duration of the surfactant/polymer flood itself, 1.5 PV, compared to the chase waterflood, ≥50 PV. Given the relatively short injection time, a possibility is thus the lack of time the surfactant has to enter the low porosity regions in order to aid the recovery of the oil within these layers. In fact, examining the results from the tracer test, Figure 4, following the injection of 1.5 PV, it is evident that the tracer is yet to enter the low porosity region at the top of the core and is slowly entering the low porosity region at the bottom of the core. Analogously, it is possible that the injected surfactant behaved similarly and effectively bypassed the low porosity regions. To best investigate this, Figure 11 provides a link between the tracer/brine test from Figure 4 and the surfactant/polymer flood recoveries themselves.

Here, curves for the temporal evolution of the average concentration of tracer (left panel, “step-up”) and brine (right panel, “step-down”) within each layer for the tracer-brine test are mapped to the associated incremental oil recovery from the surfactant/polymer flood. To aid visualization, the layers corresponding to the top two and bottom two incremental oil recoveries are shown as larger and nontransparent symbols. Evident from Figure 11 is the strong relationship between the incremental oil recovery and the rate of tracer uptake in the layers. The layers with the lowest recovery are those with the slowest tracer uptake, and the layers with the highest recovery are those with the fastest tracer uptake. We can also observe strong similarities in the step-up and step-down concentration profiles. It is thus possible that the injected surfactant entered the high porosity regions effectively, as did the tracer, and recovered most of the trapped oil, seen also in the green regions in Figure 10. However, given that the surfactant was injected for only 1.5 PV and then followed by the injection of brine in the chase waterflood, the surfactant could have been swept away before entering the low porosity regions completely. The difference between the recovery following the chase waterflood between the two low porosity regions could also be attributed to this. Examining Figure 4, the low porosity region at the bottom of the core has considerably more tracer ingress than that at the top of the core. As such, it could be expected that some surfactant also entered this region and ultimately aided the recovery of oil, albeit at a slower time scale than the remaining regions of the core.

## 5 Conclusions

Within this work, we conducted a surfactant/polymer flood in a tertiary recovery format on a Nugget sandstone core imaged with X-ray CT. The core was heterogeneous in nature and characterized by stratified layers of comparatively high and low porosity. Employing X-ray CT imaging, a spatial map of the porosity within the core was obtained, and via a step tracer test, the flow within the sample was then characterized. Through this, both a preferential flow occurring within the central, comparatively high porosity layers and a slow ingress into the low porosity layers, occurring over a time frame of 6–10 PV, were identified. This behavior was validated in the reverse tracer test: brine displacing tracer. Overall, the recovery steps implemented, waterflood, surfactant/polymer flood, and chase waterflood, yielded a combined ultimate recovery of approximately 80%. Through the use of direct imaging and comparison to an analogous experiment completed in a homogeneous sample, however, the following were observed.

- Within the surfactant/polymer flood, no oil bank was formed despite the formulation’s proven capability to do so.^22^
- The initial portion of the surfactant/polymer flood appeared to displace additional oil into the low porosity layers, which then became unrecoverable.
- Unlike the homogeneous sample, the recovery varied greatly in the transverse direction, owing to the layered nature of the sample. Specifically, low porosity layers displayed a negligible layer-averaged recovery in the surfactant/polymer flood despite the high viscosity of the injected solution. Local variations of saturation along the layers were observed though.

Overall, although the absolute recovery of the injection scheme can be largely deemed successful, considering the aforementioned observations obtained via X-ray CT imaging, there are some clear shortcomings. Most obvious is the apparent negative impact the surfactant/polymer flood had within the low porosity regions, with an increase in oil saturation. The possibility of a negative impact from a surfactant flood is not a novelty^58^; however, in the system here considered, this was not fully expected given the success of the formulation in a homogeneous system. As such, as the fluid/rock system achieved suitable recoveries overall, it is possible that a better selection of the injection scheme itself could have further improved the efficiency of the process.^59,60^ Given its flexibility, we suggest future work continue to implement direct imaging in more diverse applications to gain unique insights and provide a more complete overview of what can be considered an experimental success, possibly aiding the development of both chemical formulations and also injection schemes.^61^ Results at a core scale can be hard to translate to a reservoir scale; however, when considering the strong influence the low porosity layer displayed, and given well logs can fail in identifying thin strata,^62^ accounting for outcomes in the presence of low porosity regions can prove just as important as accounting for high porosity thief zones when performing any subsurface uncertainty risk management.

## Supporting Information Available

Permeability calculation; X-ray CT error computations; layer permeability estimation; and pressure drop measurements (PDF)
