# Supplementary material for: Porosity-Limited Transport during Two-Phase Surfactant/Polymer Floods in a Layered Sandstone
Source: Energy Fuels. 2025 Jan 23;39(5):2471–81. doi: 10.1021/acs.energyfuels.4c04866 (PMC11808638; doi:10.1021/acs.energyfuels.4c04866)
Supplement: Supplementary file 1 — ef4c04866_si_001.pdf [file ef4c04866_si_001.pdf]

Supporting Information for 'Porosity-Limited Transport during Two-Phase Surfactant/Polymer Floods in a Layered Sandstone'

Andrea Rovelli,[†] Takeshi Kurotori,[†] James Brodie,[‡] Bilal Rashid,[‡] Weparn J. Tay,[‡] and Ronny Pini[*,†]

†*Department of Chemical Engineering, Imperial College London, South Kensington SW7 2AZ, United Kingdom*

‡*BP International Ltd, Chertsey Road, Sunbury-on-Thames TW16 7LN, UK*

E-mail: r.pini@imperial.ac.uk

Introduction

Associated supporting information for main text. Includes additional details for the permeability calculation, complete calculation of the error handling of the X-ray CT images, overview of estimating the layer permeabilities and the pressure drop profiles for the waterflood and surfactant/polymer flood.

Permeability estimation

The permeability for the core sample is estimated through a multi-rate steady-state test with brine and analysed with Darcy's law:

$$q = \frac{K \Delta P}{\mu L} \tag{1}$$

Figure S1 presents both the measurement of the pressure with the changes in flowrate and the calculation of the permeability itself through the re-arrangement of Equation (1).

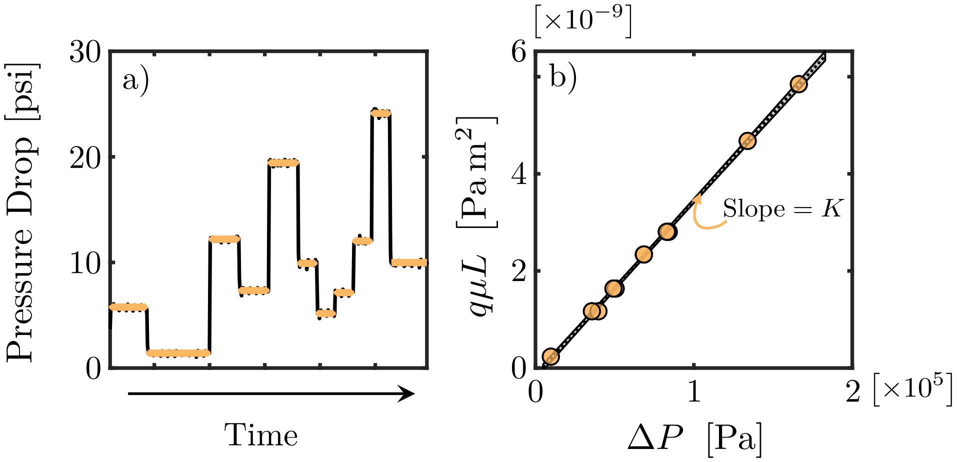

Figure S1: Pressure drop (a) and associated calculation for permeability (b) for the Nugget sandstone. Also shown in (b) is the associated 95 % confidence interval for the calculated permeability. From the slope of (b) the permeability is 36.51 mD ±0.82.

Via the slope, the permeability was calculated as 36.51 mD ±0.82, error associated with 95 % confidence interval of the linear fit.

Error propagation and correlation

To quantify and manage error an approach similar to that of Pini et al.[1] was used. For this, differences in repeated scans were calculated and this error was then propagated using equations derived from the propagation of variances[2] and given in the main text. The approach is identical to that of our previous work[3] and results given in Table S1.

Table S1: Errors in porosity, saturation and tracer concentration for differing re-sampling schemes tested. Reported are both the errors associated with three-dimensional and two-dimensional reconstructions - first and second column under each voxel size considered respectively.

	$(0.98 \times 0.98 \times 1)\text{mm}^3$		$(1.95 \times 1.95 \times 1)\text{mm}^3$		$(1.95 \times 1.95 \times 2)\text{mm}^3$		$(2.93 \times 2.93 \times 3)\text{mm}^3$	
σ_ϕ	1.7×10^{-2}	3.7×10^{-3}	1.1×10^{-2}	2.8×10^{-3}	9.6×10^{-3}	2.5×10^{-3}	6.0×10^{-3}	2.3×10^{-3}
$\sigma_{\text{S}_\text{o}}$	0.530	0.115	0.343	0.087	0.298	0.077	0.185	0.073
$\sigma_{\text{c}_\text{NaI}}$	0.292	0.064	0.188	0.048	0.164	0.042	0.101	0.040

Here σ_ϕ, $\sigma_{\text{S}_\text{o}}$ and $\sigma_{\text{c}_\text{NaI}}$ are the absolute errors in the porosity, saturation and tracer concentrations respectively.

Layer permeability estimate

In order to estimate the layer permeability from the step tracer test we can consider the following. One can estimate a layer effective velocity, v_i, as:

$$v_i = \frac{L}{t_i^{50}} \quad (2)$$

where L is the core, or layer, length and t_i^{50} is the median arrival time of tracer in each layer - obtainable from the internal concentration dataset. These effective layer velocities, coupled with the core averaged Darcy's law (Equation (1)) and the knowledge that the pressure drop along the core is equal to that along the layers, allow us to express the following:

$$\frac{v_i}{\bar{v}} = \frac{k_i}{\bar{k}} \quad (3)$$

where $\bar{v}$ is the average core interstitial velocity - known - and $\bar{k}$ is the core permeability - also known. As such, re-arranging Equation (3), an estimate for the permeability of each layer, k_i, can be made.

Also used in the main text were two models used to estimate permeability from the porosity. These were the Kozeny-Carman:[4]

$$k_i = S\frac{\phi^{\mathrm{n}}}{(1-\phi)^2} \quad (4)$$

and the Fractal-Geometry:[5]

$$k_i = S\left[6.2\phi + 1493\phi^2 + 58(10\phi)^{10}\right] \quad (5)$$

both models utilise a shape factor, S, which, in this work, was used to ensure that, for the mean porosity of the core, the permeability calculated was equal to the measured porosity

for the core. The Kozeny-Carman model utilised here also includes a variable exponent, n, which, as was done in the main text, can be used to further investigate the strength of the porosity-permeability relationship.

Pressure drop measurements

The pressure drop profiles for both the waterflood and the surfactant/polymer flood are given in Figure S2.

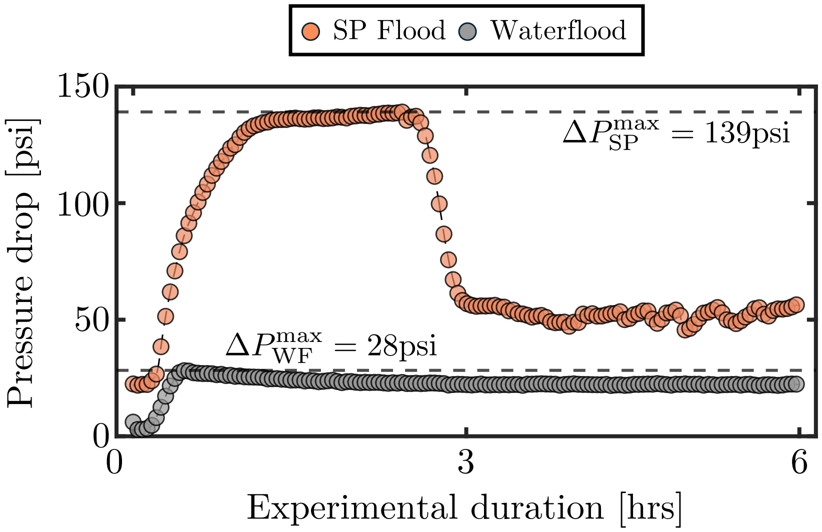

Figure S2: Pressure drop profiles for the surfactant/polymer (SP) flood and waterflood (WF). Also given are the maximum pressure drops achieved during the injection stages.

The maximum pressure drop in the two injections steps were 28 psi and 139 psi in the waterflood and surfactant/polymer flood respectively - yielding a ratio of 4.92. Important to note is that the surfactant/polymer flood pressure drop profile also includes the subsequent waterflood - starting from approximately just before 3 h. The plateau in the surfactant/polymer flood can be associated to the breakthrough of the injected fluids and flow through the preferential flow paths - comparatively high permeability layers. Once the subsequent waterflood is commenced, the pressure drop significantly decreases.

References

(1) Pini, R.; Krevor, S. C.; Benson, S. M. Capillary pressure and heterogeneity for the CO_2/water system in sandstone rocks at reservoir conditions. *Advances in Water Resources* **2012**, *38*, 48–59.

(2) Rouaud, M. *Probability, Statistics and Estimation: Propagation of Uncertainties in Experimental Measurement*; 2013.

(3) Rovelli, A.; Brodie, J.; Rashid, B.; Tay, W. J.; Pini, R. Effects of Core Size and Surfactant Choice on Fluid Saturation Development in Surfactant/Polymer Corefloods. *Energy & Fuels* **2024**, *38*, 2844–2854.

(4) Krause, M. Modeling Permeability Distributions in a Sandstone Core for History Matching Coreflood Experiments. *SPE Journal* **2011**,

(5) Pape, H.; Clauser, C.; Iffland, J. Permeability prediction based on fractal pore-space geometry. *Geophysics* **1999**, *64*, 1447–1460.
